# Expression and Co-Expression Patterns of Selected MicroRNAs and ADAM Genes in Colorectal Cancer

**DOI:** 10.3390/ijms27219983

**Published:** 2026-11-07

**Authors:** Agnieszka Kalita, Magdalena Sikora-Skrabaka, Karolina Gołąbek, Krzysztof Biernacki, Joanna Katarzyna Strzelczyk, Dariusz Waniczek, Ewa Nowakowska-Zajdel

**Affiliations:** 1 Department of Metabolic Disease Prevention, Faculty of Public Health in Bytom, Medical University of Silesia in Katowice, 40-055 Katowice, Poland; 2 Department of Clinical Oncology, No. 4 Provincial Specialist Hospital, 41-902 Bytom, Poland; 3 Department of Medical and Molecular Biology, Faculty of Medical Sciences in Zabrze, Medical University of Silesia in Katowice, 41-808 Zabrze, Poland; 4 Department of Oncological Surgery, Faculty of Medical Sciences in Zabrze, Medical University of Silesia in Katowice, 41-514 Katowice, Poland

**Keywords:** colorectal cancer, microRNA, miR-125b-5p, miR-338-3p, miR-30c-5p, *ADAM10*, *ADAM12*, *ADAM17*, *ADAM28*, gene expression

## Abstract

Colorectal cancer (CRC) is a leading cause of cancer-related death worldwide, and its molecular pathogenesis is not fully understood. This exploratory study investigated the expression of three selected microRNAs in colorectal cancer and their associations with previously characterized ADAM transcripts. RT-qPCR was used to assess miR-125b-5p, miR-338-3p, and miR-30c-5p in paired tumor and histologically tumor-free surgical margin tissues from 67 patients. Previously obtained *ADAM10*, *ADAM12*, *ADAM17*, and *ADAM28* transcript data from the same cohort were included in correlation analyses. The Wilcoxon signed-rank test and Spearman’s rank correlation were applied, with Benjamini–Hochberg adjustment for multiple testing. MiR-125b-5p expression was lower in tumors than in matched margins (median 0.46 versus 1.33; adjusted *p* = 0.035). No significant paired differences were identified for miR-338-3p or miR-30c-5p. Margin miR-125b-5p expression was associated with neoadjuvant-treatment history (adjusted *p* = 0.033), although the treated subgroup was small. No miRNA-ADAM transcript correlations remained significant after adjustment. Positive correlations among some miRNA pairs were observed within tumor and margin tissues. The reduced level of relative miR-125b-5p expression in colorectal cancers is an observational finding consistent with the tumor-suppressor functions described in the literature, but it does not constitute proof of those functions. Inter-miRNA correlations do not demonstrate shared regulatory mechanisms, and the absence of miRNA-ADAM transcript associations does not exclude post-transcriptional regulation.

## 1. Introduction

Colorectal cancer (CRC) is the second leading cause of cancer-related deaths worldwide and the most common malignant tumor among cancers of the digestive system [1]. It represents a substantial global health burden, with considerable geographic variation in incidence and mortality [2].

Numerous studies are currently underway to investigate the pathogenesis and progression of CRC, as well as methods for its early detection and treatment. Adamalysines (ADAM family proteins) are among the potential factors involved in the pathogenesis and development of CRC. ADAM proteins are a group of glycoproteins that combine the functions of proteases and adhesion proteins [3]. They are involved in many pathways responsible for the onset and progression of CRC, such as the Notch, Epidermal Growth Factor (EGF), Wnt or Insulin-Like Growth Factor (IGF) signaling [4,5,6].

MicroRNAs (miRNAs) have emerged as important regulators in many cancer development pathways. MiRNAs are short, approximately 22-nucleotide, non-coding RNAs that regulate gene expression at the post-transcriptional level. Initially, within the cell nucleus and cytoplasm, the enzymes Drosha and Dicer convert the long primary miRNA transcript first into a hairpin-structured precursor and then into a mature molecule, which is incorporated into the RISC protein complex with an Argonaute core. Next, the RISC complex, armed with miRNA, recognizes a complementary segment of messenger RNA—usually in the 3′UTR region—*via* a short seed sequence, allowing a single miRNA to regulate many different transcripts simultaneously [7]. Consequently, miRNAs function as components of complex regulatory networks involved in proliferation, cell differentiation, apoptosis, cellular metabolism or stress responses [8]. Depending on the genes they regulate and the cellular context, miRNAs can function as oncogenes (oncomiRNAs) or tumor suppressors [9]. A good example of the dual function of miRNA is miR-125b itself, which acts as an oncogene in hematologic cancers, such as acute myeloid leukemia or acute lymphoblastic leukemia, a fact confirmed experimentally in a mouse model of leukemia induced by its overexpression. Conversely, in breast, hepatocellular, and ovarian cancers, it acts as a tumor suppressor, regulating other target genes depending on the type of cancer [10,11]. Furthermore, circulating miRNAs also mediate bidirectional communication between cancer cells and components of the tumor microenvironment, including cancer-associated fibroblasts (CAFs) and tumor-associated macrophages (TAMs), reprogramming their metabolism and polarization in a way that promotes tumor progression, immunosuppression, and chemoresistance [12].

Among the best-studied and most thoroughly characterized miRNAs in CRC are miR-21, the miR-34 family, miR-143/145, miR-155, miR-135a/b, miR-17-92, and the miR-200 family [13]. They are involved in the regulation of signaling pathways such as the Wnt/β-catenin, PI3K/AKT, RAS/MAPK, Transforming Growth Factor (TGF-β), p53, and NF-κB pathways, as well as processes related to epithelial–mesenchymal transition (EMT) [14]. One of the best-characterized oncogenic miRNAs in CRC is miR-21. Its increased expression is associated with the development and progression of CRC, which occurs, among other mechanisms, through the inhibition of the expression of tumor suppressor genes such as *PTEN* and *PDCD4* and the regulation of the PI3K/AKT, MAPK, and Wnt/β-catenin pathways [15]. In contrast, miR-34a, whose expression is regulated by p53, exhibits tumor-suppressive properties by regulating genes associated with cell proliferation, survival, and apoptosis [16].

Among the numerous miRNAs, miR-125b-5p, miR-338-3p, and miR-30c-5p have also been the subject of studies suggesting their involvement in the development of CRC. MiR-125b-5p has been reported to have both suppressor and oncogenic effects [17]. It has been shown to play a role in regulating the proliferation, apoptosis, migration, and invasion of CRC cells by interacting with, among others, *MCL1*, *CFTR*, and *CGN*, as well as influencing the *EMT* and the RhoA/ROCK pathway. Furthermore, the involvement of the miR-125b-5p/TRAF6 axis in regulating the migration and invasion of CRC cells has been described, indicating the complex role of this miRNA in tumor progression [18,19]. Additionally, miR-125b-5p inhibits proliferation and induces apoptosis in CRC cells by silencing *STAT3*, and its low tissue levels correlate with the presence of liver metastases and shorter disease-free survival [20]. MiR-338-3p is most commonly described as a tumor-suppressor miRNA in CRC. Studies of tumor tissues have shown reduced expression of this miRNA compared to adjacent tissue, and lower levels have been associated with more advanced disease stages, depth of invasion, lymph node involvement, and the presence of metastases. Functionally, miR-338-3p can inhibit the proliferation, migration, and invasion of cancer cells, in part through direct regulation of *MACC1*, a gene strongly associated with CRC progression and metastasis [21]. *SMO*, a member of the Hedgehog pathway, has also been identified as a potential target of miR-338-3p [22]. miR-30c-5p belongs to the miR-30 family and is involved in regulating processes related to proliferation, tumor stem cell properties, and the response to CRC treatment. Experimental studies have demonstrated reduced expression of the miR-30-5p family in CRC tissues and cell lines, whereas increasing its levels limited proliferation, spheroid formation, and the stemness properties of cancer cells. One of the key mechanisms of action involves the regulation of *USP22* and the subsequent inhibition of the Wnt/β-catenin pathway, including a reduction in β-catenin activity and the expression of its target genes, such as *MYC* and *AXIN2* [23]. MiR-30c-5p also participates in the regulation of the circ3823/miR-30c-5p/TCF7 axis, which is associated with proliferation, angiogenesis, and metastasis in CRC [24]. Together, these miRNAs encompass most of the key oncogenic pathways in CRC, representing an important yet incompletely understood, component of the molecular regulation of this cancer.

Published studies indicate that certain ADAM-family transcripts can be regulated by miRNAs in specific experimental settings. For example, miR-338-3p-mediated regulation of ADAM17 has been described in gastric cancer [25,26], whereas miR-30c has been reported to regulate ADAM19 in colorectal cancer [27]. These findings provide a biological rationale for examining candidate miRNA-ADAM relationships, but they do not establish equivalent interactions in CRC.

The primary aim of the present study was to characterize the relative expression of miR-125b-5p, miR-338-3p, and miR-30c-5p in paired colorectal tumor and histologically tumor-free surgical margin tissues. We additionally examined associations with *ADAM10*, *ADAM12*, *ADAM17*, and *ADAM28* transcript levels previously determined in the same patient cohort [28]. Exploratory secondary analyses addressed inter-miRNA correlations and relationships with selected clinicopathological characteristics. Thus, unlike our earlier ADAM-focused publication, the present study reports newly measured miRNA expression data and evaluates tissue-level miRNA-ADAM transcript associations; it was not designed to demonstrate direct molecular targeting. The study protocol was approved by the Bioethics Committee of the Medical University of Silesia in Katowice, Poland (approval number PCN/0022/KB1/42/VI/14/16/18/29/20).

## 2. Results

New miRNA expression measurements were obtained from tissue samples collected from 33 men and 34 women. *ADAM10*, *ADAM12*, *ADAM17*, and *ADAM28* transcript expression values were retrieved from our previous study of the same cohort [28] and were used here only for the planned cross-marker association analyses.

In the paired tissue analysis across the study cohort, the relative expression levels of the three selected miRNAs were evaluated between tumor tissue and matched surgical margins. A statistically significant difference in expression was demonstrated for miR-125b-5p after Benjamini–Hochberg correction. Specifically, the relative expression of miR-125b-5p was significantly lower in tumor tissue compared to matched surgical margins (median 0.46, IQR 1.41 vs. median 1.33, IQR 2.35, *n* = 66, *p* = 0.0099, *p_adj_* = 0.035, *r* = 0.225). For miR-30c-5p and miR-338-3p, expression was nominally lower in tumor tissue than in matched margins, but this difference was not statistically significant. The expression parameters for *ADAM* family genes were characterized in our previous publication, and the miRNA expression results for the current cohort are summarized in Table 1.

**Figure 1:**
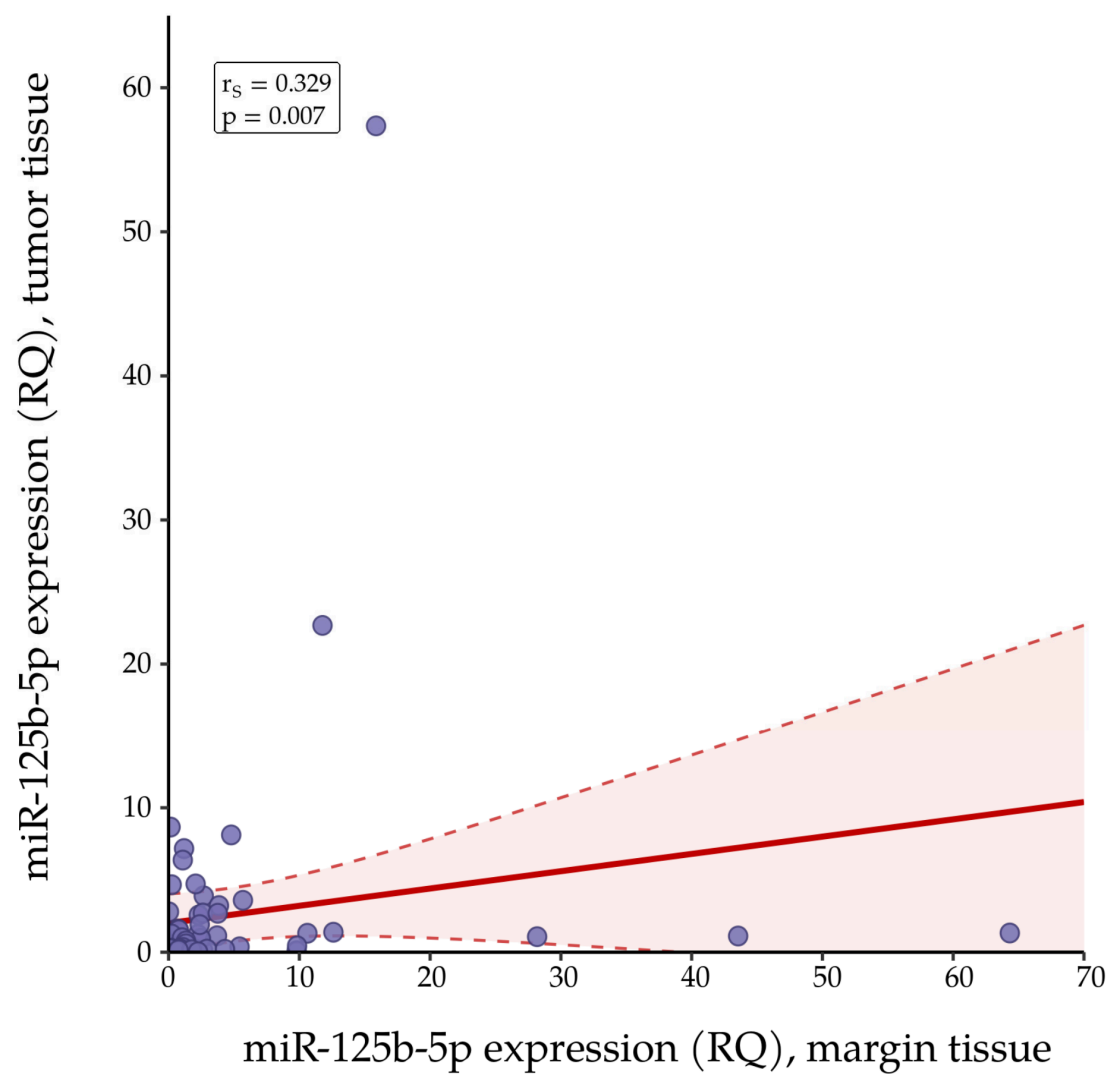
Correlation between relative expression (RQ) of miR-125b-5p in colorectal tumor tissue and in the matched margin tissue of the same patient. Each point represents one patient (purple fill: paired tumor-vs-margin comparison). The solid line shows the fitted linear regression with its 95% confidence interval (shaded ribbon bounded by dashed lines).

In addition, a statistically significant positive correlation in expression was observed for miR-125b-5p between tumor tissue and matched surgical margins in the same patient (r_S_ = 0.33, *n* = 66, *p_adj_* = 0.011, Figure 1). No statistically significant correlations between tumor and margin tissue were observed for the remaining miRNAs (miR-338-3p and miR-30c-5p).

We also evaluated differences in the expression of selected miRNAs depending on sex across unpaired tissue samples. MiR-125b-5p expression in tumor tissue was nominally higher in men than in women (median 1.10 vs. 0.25, *p* = 0.007, *p_adj_* = 0.060). Similarly, in surgical margin tissue, miR-30c-5p expression was nominally higher in men than in women (median 1.67 vs. 0.94, *p* = 0.012, *p_adj_* = 0.099). The remaining unpaired subgroup differences were not statistically significant.

When the paired tumor-versus-margin analysis of miR-125b-5p was stratified by sex, a nominal difference was most pronounced in women (*n* = 32, *p* = 0.0016, *p_adj_* = 0.161), while no nominal paired difference was detected in men (*n* = 33, *p* = 0.393, *p_adj_* = 0.665). However, no sex-specific differences in paired tissue comparisons remained statistically significant after Benjamini–Hochberg correction. These subgroup findings should therefore be interpreted strictly as exploratory and hypothesis-generating.

No statistically significant differences in the expression of selected miRNAs were observed based on tumor grade, pathomorphological classification (T, N, M parameters), or clinical stage.

Statistical analyses of the expression of selected miRNAs and *ADAM* genes were also performed based on tumor location, comparing right-sided versus left-sided tumors as well as rectal versus non-rectal tumors. Baseline *ADAM* genes expression results were described in our previous study, and here they were evaluated with respect to location-specific patterns alongside the miRNA expression profiles.

In surgical margin tissue, a nominal variation in miR-125b-5p expression was observed across specific colon segments, with higher median values in the ascending colon compared to other locations (median 5.69, *n* = 7 vs. medians 0.74–1.73, *p* = 0.004–0.049). A similar nominal pattern was noted in tumor tissue (median 1.23 vs. 0.15, *p* = 0.045). However, due to small subgroup sizes, these location-specific differences did not maintain statistical significance following Benjamini–Hochberg correction (*p_adj_* ≥ 0.199).

In paired tumor-versus-margin comparisons stratified by location, miR-125b-5p expression in non-rectal colon tumors showed a nominal decrease in tumor tissue compared to matched margins (*n* = 44, median 0.45 vs. 1.03, *p* = 0.036, *p_adj_* = 0.200). Nominal paired differences were also observed for *ADAM12* in right-sided tumors (*n* = 20, median 1.34 in tumor vs. 0.18 in margin, *p* = 0.016, *p_adj_* = 0.161) and non-rectal colon tumors (*n* = 40, median 1.10 vs. 0.23, *p* = 0.034, *p_adj_* = 0.197). However, after Benjamini–Hochberg adjustment, no differences in expression based on tumor location remained statistically significant for any of the investigated miRNAs or *ADAM* genes (all *p_adj_* ≥ 0.161).

No significant differences in the expression of selected miRNAs were observed among patients based on age (≤65 years vs. >65 years) or substance use disorders, including nicotine dependency (i.e., patients consuming 10 or more packs of cigarettes each year and currently smoking or quit smoking within the past 15 years) and alcohol dependency (i.e., patients consuming 2.86 g or more of ethanol each day or approximately two alcoholic beverages per week).

In the non-obese subgroup (BMI < 30, *n* = 48), a nominal trend toward lower expression of miR-125b-5p was observed in tumor tissue compared with matched surgical margins (median 0.41 vs. 1.26, *p* = 0.022, *p_adj_* = 0.168). No other trend related to BMI categories reached statistical significance after Benjamini–Hochberg adjustment (all *p_adj_* ≥ 0.161).

No statistically significant differences in miRNA expression were observed in tumor or margin tissue between patients with and without concomitant cardiovascular diseases (CVD). In patients with comorbid CVD and valid paired tissue measurements (*n* = 45), miR-125b-5p expression showed a nominal difference between tumor tissue and matched margins (median 0.33 vs. 1.33, *p* = 0.009), which was not statistically significant after correction (*p_adj_* = 0.161).

Notably, miR-125b-5p expression in surgical margin tissue differed statistically between patients who had undergone neoadjuvant cancer treatment prior to surgery and those who had not (*n* = 11 vs. 53, median 3.71 vs. 1.04, *p* = 0.0037, *p_adj_* = 0.033). In paired tumor-versus-margin comparisons within the neoadjuvant group (*n* = 11), miR-125b-5p expression was lower in tumor tissue (median 0.95 vs. 3.71, *p* = 0.045, *p_adj_* = 0.222). However, because only 11 patients in the cohort received neoadjuvant therapy, these observations should be interpreted with caution and regarded as exploratory.

**Figure 2:**
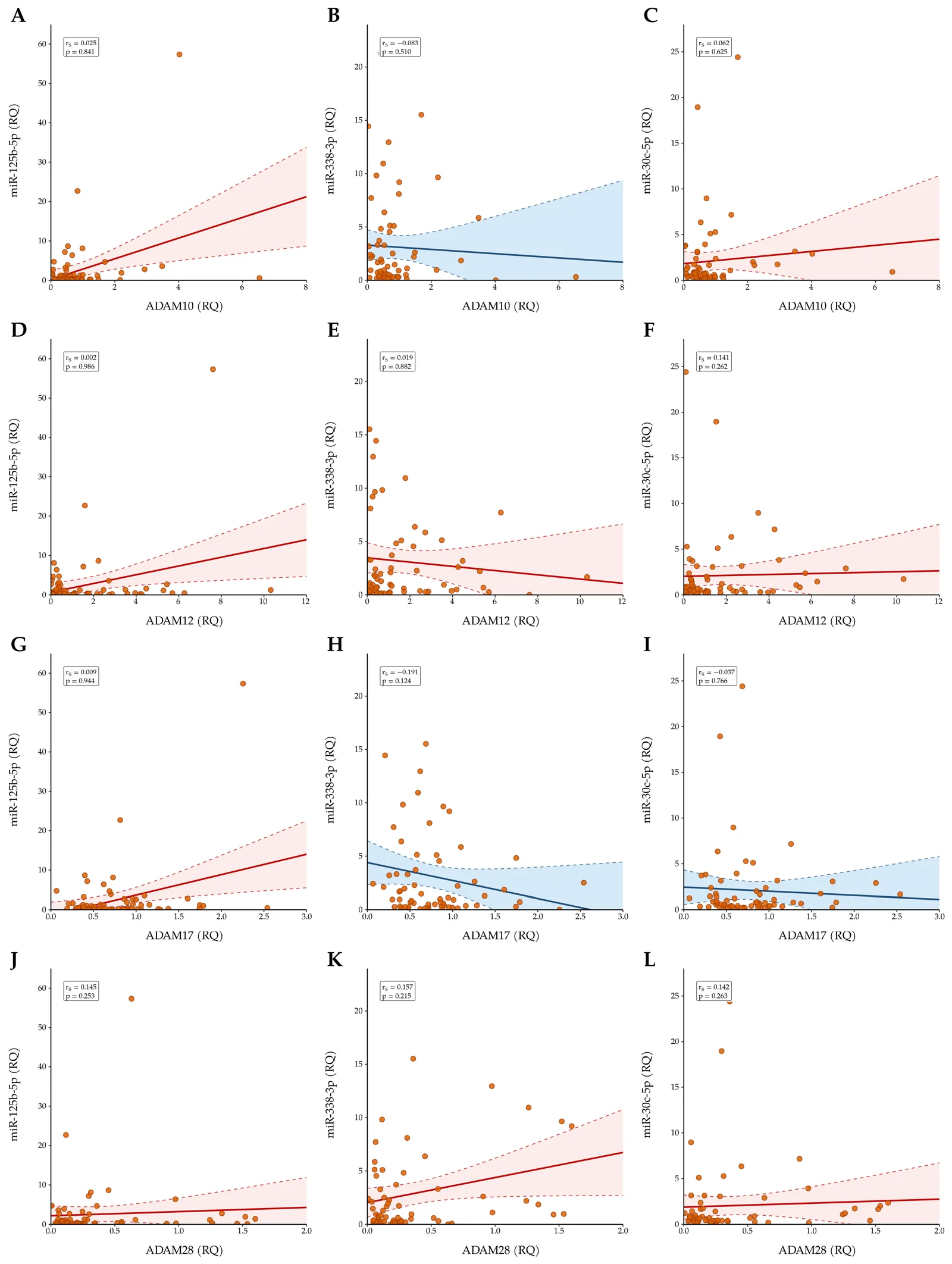
Scatter plots of the relative expression (RQ) of selected miRNAs versus *ADAM* genes in colorectal tumor tissue: (**A**) miR-125b-5p vs. *ADAM10*; (**B**) miR-338-3p vs. *ADAM10*; (**C**) miR-30c-5p vs. *ADAM10*; (**D**) miR-125b-5p vs. *ADAM12*; (**E**) miR-338-3p vs. *ADAM12*; (**F**) miR-30c-5p vs. *ADAM12*; (**G**) miR-125b-5p vs. *ADAM17*; (**H**) miR-338-3p vs. *ADAM17*; (**I**) miR-30c-5p vs. *ADAM17*; (**J**) miR-125b-5p vs. *ADAM28*; (**K**) miR-338-3p vs. *ADAM28*; (**L**) miR-30c-5p vs. *ADAM28*. Solid lines show the fitted linear regression, shaded ribbons bounded by dashed lines show the 95% confidence interval. Line color indicates the sign of the fitted slope (red—positive and blue—negative).

**Figure 3:**
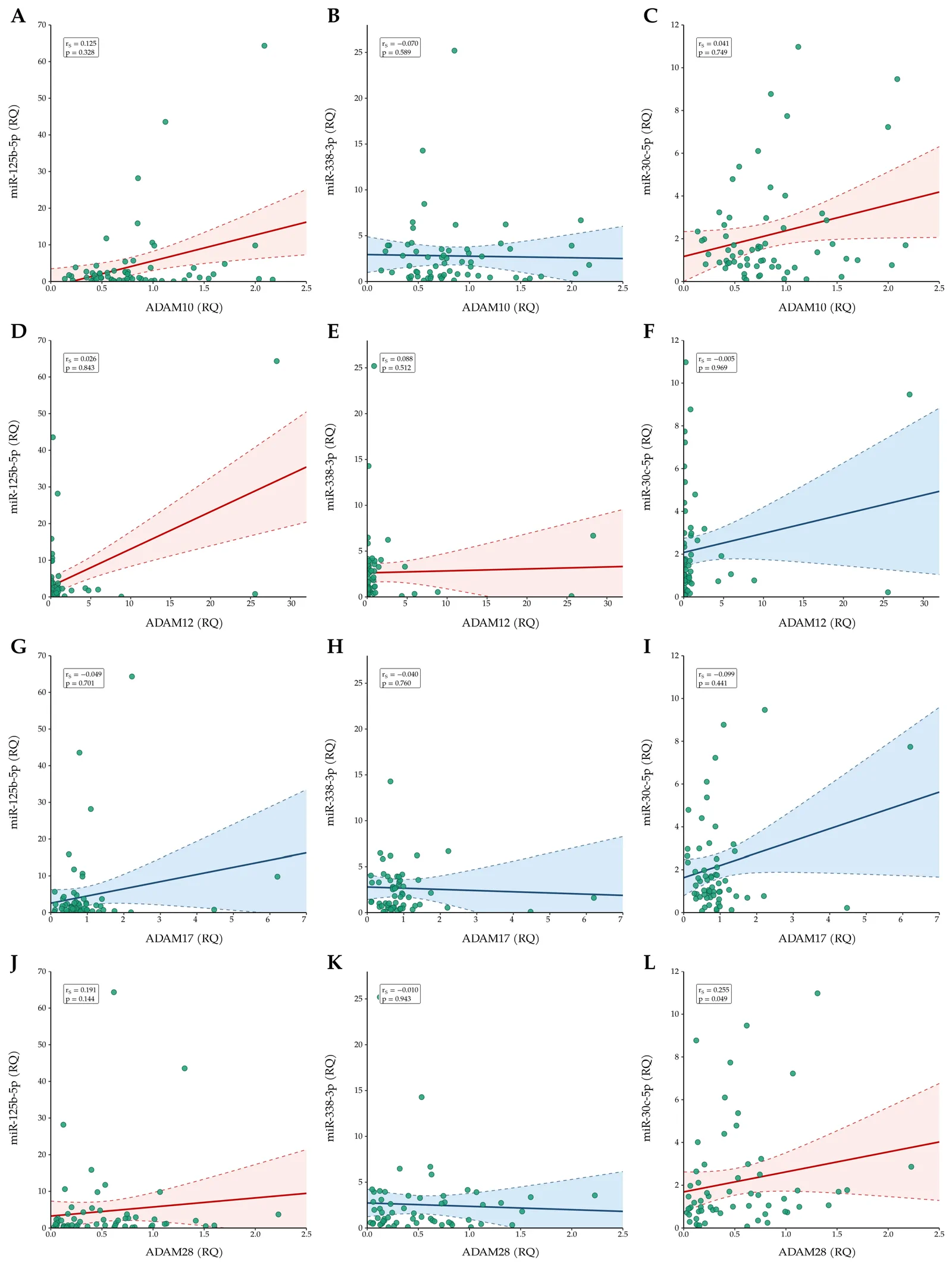
Scatter plots of the relative expression (RQ) of selected miRNAs versus *ADAM* genes in matched margin tissue: (**A**) miR-125b-5p vs. *ADAM10*; (**B**) miR-338-3p vs. *ADAM10*; (**C**) miR-30c-5p vs. *ADAM10*; (**D**) miR-125b-5p vs. *ADAM12*; (**E**) miR-338-3p vs. *ADAM12*; (**F**) miR-30c-5p vs. *ADAM12*; (**G**) miR-125b-5p vs. *ADAM17*; (**H**) miR-338-3p vs. *ADAM17*; (**I**) miR-30c-5p vs. *ADAM17*; (**J**) miR-125b-5p vs. *ADAM28*; (**K**) miR-338-3p vs. *ADAM28*; (**L**) miR-30c-5p vs. *ADAM28*. Solid lines show the fitted linear regression, shaded ribbons bounded by dashed lines show the 95% confidence interval. Line color indicates the sign of the fitted slope (red—positive and blue—negative).

No statistically significant correlations in expression were found between the investigated miRNAs and *ADAM* genes mRNA levels in either tumor tissue or matched surgical margins after multiple testing adjustment. A nominal positive correlation between miR-30c-5p and ADAM28 expression in surgical margin tissue (*p* = 0.049) did not remain significant after Benjamini–Hochberg correction (*p_adj_* = 0.305) and is therefore not interpreted as an association. The results are presented in Table 2 and Table 3, and in Figure 2 and Figure 3.

**Figure 4:**
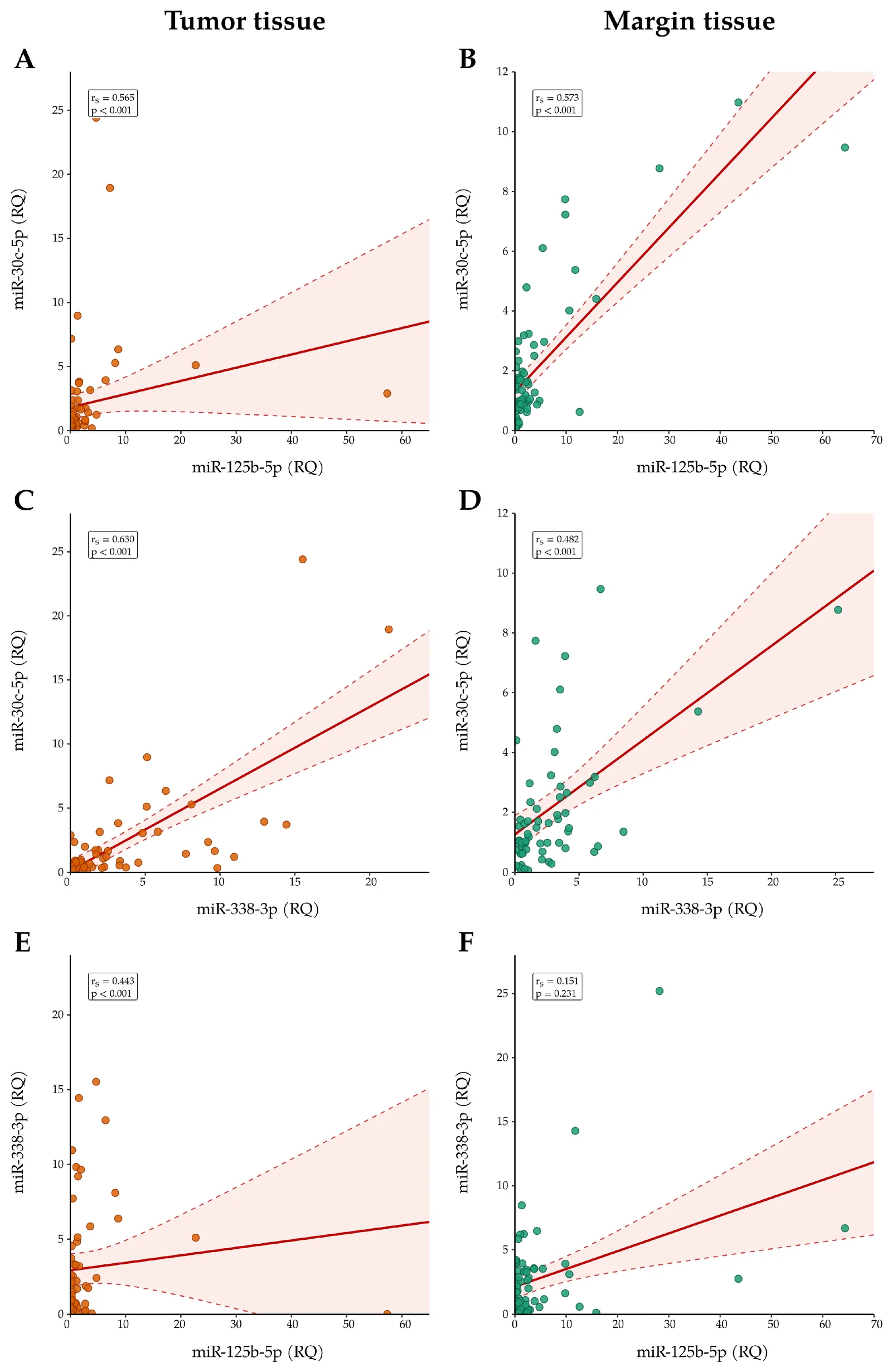
Correlations between the relative expression (RQ) of selected miRNAs in tumor and matched margin tissue of the same patients: (**A**) miR-30c-5p vs. miR-125b-5p in tumor tissue (orange points); (**B**) miR-30c-5p vs. miR-125b-5p in matched margin tissue (teal points); (**C**) miR-30c-5p vs. miR-338-3p in tumor tissue (orange points); (**D**) miR-30c-5p vs. miR-338-3p in matched margin tissue (teal points); (**E**) miR-338-3p vs. miR-125b-5p in tumor tissue (orange points); (**F**) miR-338-3p vs. miR-125b-5p in matched margin tissue (teal points). Solid lines show fitted linear regression with 95% confidence intervals (shaded ribbons bounded by dashed lines).

Significant positive co-expression patterns were demonstrated between all investigated miRNA pairs in tumor tissue (r_S_ = 0.44–0.63, *p_adj_* < 0.002). Similarly, significant inter-miRNA correlations were observed in surgical margin tissue for the miR-125b-5p-miR-30c-5p and miR-338-3p-miR-30c-5p pairs (r_S_ = 0.48–0.57, *p_adj_* < 0.001), but not for miR-125b-5p-miR-338-3p (r_S_ = 0.15, *p_adj_* = 0.661). The detailed co-expression matrix and correlation results are presented in Table 4 and Figure 4.

## 3. Discussion

This study extends our previous investigations of ADAM-family genes and proteins in colorectal cancer [28,29,30,31,32] by presenting newly generated expression data for miR-125b-5p, miR-338-3p, and miR-30c-5p in patient-matched tumor and histologically nonmalignant surgical margin tissues. The *ADAM10*, *ADAM12*, *ADAM17*, and *ADAM28* transcript results were published previously for the same cohort [28]; their inclusion here made it possible to examine previously unreported associations with miRNA expression. The central new observations were lower relative miR-125b-5p expression in tumor tissue, positive correlations among selected normalized miRNA expression measurements, and a lack of statistically significant miRNA-ADAM transcript associations after multiple-testing adjustment. These tissue-level associations should be interpreted as exploratory and do not establish direct post-transcriptional regulation.

A key finding of this study is the significant positive co-expression observed between the analyzed miRNAs in both tumor tissue and surgical margin tissue from the same patient, which persisted after BH adjustment. In tumor tissue, all three miRNA pairs exhibited significant co-expression, whereas in margin tissue significant co-expression was observed for miR-125b-5p-miR-30c-5p and miR-338-3p-miR-30c-5p. The observation that several miRNA pairs exhibited coordinated co-expression in both tumor and adjacent tissue, may reflect shared biological regulation; however, because all miRNAs were normalized to the same reference miRNA and calibrator, shared technical variation may also have contributed to these correlations. In addition, miR-125b-5p expression in tumor tissue correlated with its expression in the matched margin tissue of the same patient (r_S_ = 0.33, *p_adj_* = 0.011), whereas no such correlation was found for the other two miRNAs. This suggests that part of the variability in miR-125b-5p expression is patient-specific and shared by both tissues, which is consistent with the notion that tumor-adjacent tissue retains molecular features related to the tumor and the host [33]. Coordinated co-expression of miRNAs has been widely described in the literature, including in CRC. Farc et al. described correlations between the expression of miR-29a, miR-146a, miR-215 and miR-449 in both colorectal tumors and adjacent normal tissues in 40 patients with CRC. They also demonstrated that the strength of the correlation between miRNAs can vary across tissue compartments, observing stronger correlations for miR-215 with miR-29a and miR-449 in tumor tissue than in normal tissue [34]. In our study, the miR-125b-5p-miR-338-3p pair exhibited significant co-expression in tumor tissue but not in margin tissue, which is consistent with the notion that relationships and correlations between individual miRNAs can be influenced by the tissue context. However, the lack of significant co-expression between miR-125b-5p and miR-338-3p in margin tissue may also indicate that tumor-associated molecular changes alter these relationships. Therefore, it is important to recognize that co-expression alone does not prove direct regulatory interactions between miRNAs, and functional experiments would be necessary to determine the mechanisms underlying these relationships. Furthermore, Li et al. used weighted gene co-expression network analysis (WGCNA) to identify modules containing mRNAs, lncRNAs, and miRNAs with differential expression based on data from 439 patients with CRC. One of the identified modules included 77 miRNAs, along with numerous lncRNAs and mRNAs, demonstrating that miRNAs may be involved in many signaling pathways and transcriptional networks in CRC [35]. Similarly, Wang et al. identified regulatory networks involving miRNAs, transcription factors, and cancer-associated genes, highlighting the complex and interconnected nature of gene regulation in CRC [36]. Although the three miRNAs analyzed in our study were not specifically evaluated in those network analyses, those findings indicate that coordinated miRNA co-expression may reflect shared molecular programs in CRC. Further studies using larger cohorts and network-based analyses are required to determine whether these associations represent reproducible miRNA co-expression modules and whether their organization differs systematically between tumor and non-tumor tissues.

In our paired tissue analysis, miR-125b-5p expression was significantly lower in tumor tissue compared to matched surgical margins (*p_adj_* = 0.035). For miR-30c-5p and miR-338-3p, expression was nominally lower in tumor tissue than in matched margins, but these differences were not statistically significant (*p* = 0.125 and *p* = 0.305, respectively). Stevens et al. addressed differences in miRNA expression between paired and unpaired groups in colorectal cancer by describing interindividual variability and demonstrating that pairing can increase statistical power compared to analyses that treat samples as independent observations [37]. Furthermore, correction using the Benjamini–Hochberg procedure reduces the likelihood of false-positive findings when multiple hypotheses are tested [38]. Therefore, these results should be viewed as exploratory rather than definitive conclusions.

Previous studies have reported varying expression patterns of miR-125b-5p, miR-338-3p and miR-30c-5p in CRC. Nishida et al. associated higher miR-125b-5p expression with larger tumor size, deeper invasion, presence of metastases, and poorer prognosis [39]. In contrast to Nishida et al., our overall cohort demonstrated a significant reduction of miR-125b-5p in tumor tissue compared to matched margins, aligning with studies reporting tumor-suppressive activity. However, no statistically significant differences in miR-125b-5p expression were detected according to clinical or pathological stage. Similarly, Chen et al. reported reduced expression of miR-125b-5p in tumor tissue, which they attributed to promoter hypermethylation [40]. This illustrates the dual oncogenic and tumor-suppressive roles of this miRNA across various malignancies, including CRC. These context-dependent differences may relate to tumor stage [18] or methodological factors, such as the choice of reference miRNA, which can vary between tumor and normal tissues and bias relative quantification [41]. Additionally, reduced expression of miR-338-3p and miR-30c-5p in tumor tissue compared to adjacent margins has been widely reported, with miR-338-3p acting as a tumor suppressor in multiple studies [21,22,23]. In our cohort, both miRNAs displayed a similar downward trend in tumor tissue, although without statistical significance. Such discrepancies across published studies are common in miRNA research, as miRNA functions in CRC appear to be highly context-dependent. Notably, surgical margin tissue adjacent to the tumor is not equivalent to healthy mucosa. Transcriptomic analyses of tumor-adjacent tissue have revealed an intermediate molecular state between normal and neoplastic tissue [33], which may obscure differences between tumor and margin compartments in a patient-specific manner. Furthermore, the outcomes of RT-qPCR-based miRNA evaluations depend heavily on the choice of reference miRNA and calibrator sample [41].

The expression of miR-125b-5p in the tumor tissue was approximately four-fold higher in men than in women (median 1.10 vs. 0.25, *p* = 0.007, *p_adj_* = 0.060), and margin miR-30c-5p levels were also higher in men (median 1.67 vs. 0.94, *p* = 0.012, *p_adj_* = 0.099). The sex-specific differences in miR-125b-5p and miR-30c-5p expression that we observed were inconsistent across paired and unpaired analyses and did not reach statistical significance after Benjamini–Hochberg adjustment. Therefore they should be considered exploratory. Although sex-dependent differences in miRNA expression have been reported in the literature, for instance, lower miR-30c-5p expression in tumor tissue was observed in male patients with lymph node involvement and associated with survival outcomes [42], our findings do not allow for definitive conclusions. Nevertheless, patient sex represents a relevant biological variable that warrants inclusion in larger cohort studies. Such sex-related differences in miRNA expression appear biologically plausible, given that CRC exhibits marked sexual dimorphism: men show higher incidence and mortality rates, whereas women, particularly premenopausal women, frequently exhibit longer overall survival and a higher proportion of right-sided tumors. Harvey and Harvey proposed that these differences may be driven by estrogen signaling mediated by estrogen receptor β (ERβ). This receptor is more strongly expressed in female colonic tissue and appears to act as a tumor suppressor, partly through the modulation of miRNA expression [43]. However, sex-dependent differences in miRNA expression are not uniform in direction across studies. For example, Hasáková et al. reported higher expression of both strands of miR-21 and of miR-16 in tumor and adjacent colonic tissue of male patients [44], indicating that the effect of sex is miRNA-specific rather than global. Possible explanations for the observed sex-related variations include hormonal regulation of miRNA transcription and sex-related differences in tumor location and molecular characteristics. However, confirming these potential mechanisms would require data on estrogen receptor expression and hormonal status in larger cohorts.

Additionally, we observed a statistically significant difference in miR-125b-5p expression in surgical margin tissue between patients who received neoadjuvant therapy prior to surgery and untreated patients (*n* = 11 vs. 53, median 3.71, IQR 6.92 vs. median 1.04, IQR 1.93, *p* = 0.0037, *p_adj_* = 0.033), whereas paired tumor-versus-margin comparisons within the neoadjuvant subgroup showed only a nominal difference (*n* = 11, median 0.95 vs. 3.71, *p* = 0.045, *p_adj_* = 0.222). The interpretation of these results is limited by the small subgroup size (*n* = 11), as well as by heterogeneity in treatment modalities, therapeutic regimens, and time intervals between neoadjuvant therapy and surgery. Furthermore, neoadjuvant treatment may affect both the tumor and the surgical margin, so the margin cannot be regarded as untreated reference tissue in these patients. Because treatment allocation was based on clinical indications, the observed difference may also reflect patient selection or other confounding factors. Therefore, this finding represents an association with neoadjuvant treatment history rather than evidence of a therapeutic effect on miRNA expression. Nevertheless, a treatment-associated alteration in miR-125b expression is biologically plausible, as described by Svoboda et al., who observed an increase in the expression of this miRNA in rectal cancer tissue following the initiation of capecitabine-based therapy [45]. This indicates that while the association between neoadjuvant treatment history and miR-125b-5p expression is statistically significant, it requires validation in larger, clinically characterized cohorts. Treatment history should be routinely considered a key confounding variable in future miRNA studies, with standardized tissue sampling at defined therapeutic time points.

No statistically significant association with tumor location was detected for either the miRNAs or *ADAM* genes under study. Nominal variations in miR-125b-5p and *ADAM12* gene expression in the ascending colon, as well as differences related to right-sided versus left-sided tumors or rectal location, were based on small subgroups and did not remain statistically significant after applying Benjamini–Hochberg correction (*p_adj_* ≥ 0.161). Therefore, these subgroup findings should be considered solely as exploratory hypothesis-generating observations. Nevertheless, an influence of location is biologically plausible, as proximal and distal colon tumors differ in their molecular, pathological, and clinical characteristics [46]. A recent study reported lower miR-185-5p levels in right-sided tumors [41]. Left-sided and right-sided tumors also exhibit distinct clinical outcomes and prognosis [47]. Therefore, tumor location should be considered when designing larger studies on miRNA and *ADAM* expression.

Despite the interactions predicted by miRCode, miRDB and TargetScan, as well as published experimental evidence for miR-338-3p-*ADAM17* regulation in gastric cancer [25,26], no statistically significant correlations were found between the expression of the selected miRNAs and the expression of the selected genes of the *ADAM* family in either tumor tissue or matched surgical margins. The only nominal trend observed in surgical margin tissue between miR-30c-5p and *ADAM28*, did not remain statistically significant after Benjamini–Hochberg adjustment. However, although a direct relationship between *ADAM* genes transcript levels and the selected miRNAs was not demonstrated at the tissue level, potential regulatory interactions cannot be definitively ruled out. Several biological and methodological factors may account for these findings. First, miRNAs frequently suppress target gene expression at the translational level without inducing marked mRNA degradation [48,49,50], whereas this study evaluated *ADAM* expression exclusively at the transcript level. Second, bulk tissue homogenization combines distinct cell types, potentially masking cell-type specific regulatory dynamics between miRNAs and *ADAM* transcripts [51,52]. Third, the investigated miRNAs were selected based on in silico predictions rather than experimentally verified target interactions in colorectal tissue. Therefore, these findings should be interpreted with caution, indicating that a direct relationship between these miRNAs and the expression of *ADAM* genes is not evident at the tissue mRNA level. To definitively establish whether these miRNAs regulate specific ADAM proteins in colorectal cancer, future investigations incorporating combined miRNA and protein-level evaluations, alongside functional cell-based assays, are required.

Several limitations of the present study should be acknowledged. Although the total study cohort comprised 67 patients, minor missing data for specific molecular markers or clinical parameters led to slightly varying sample sizes across individual paired comparisons (*n* = 57–66 pairs per marker), which may have subtly affected subgroup statistical power. Subgroup evaluations were based on small sample sizes, and following multiple testing adjustment, most subgroup differences did not maintain statistical significance (with the exception of margin miR-125b-5p expression in relation to neoadjuvant treatment history). Therefore, these findings should be regarded strictly as exploratory and hypothesis-generating. The study was not powered a priori, and subgroup analyses could detect only large effects; therefore, non-significant subgroup results should not be interpreted as evidence of no effect. Furthermore, the surgical margin tissue utilized as a control is not fully equivalent to completely healthy, uninfluenced mucosa, which may affect relative expression comparisons. Measuring expression in bulk tissue homogenatesprecludes distinguishing transcripts derived from tumor cells, stromal cells, and infiltraing immune cells. Consequently, differences in cellular composition between tissue compartments may influence both tumor-versus-margin comparisons and miRNA–*ADAM* correlation patterns. Additionally, because protein levels were not assessed and functional validation was not performed, the absence of correlation at the transcript level does not preclude post-transcriptional or translational regulation of ADAM proteins by miRNAs. Therefore, potential regulatory relationships within specific miRNA-*ADAM* pairs cannot be definitively excluded and warrant further functional investigation. The use of RNAlater, frozen storage, standardized assays, technical duplicates, negative controls, and a common pooled calibrator supported analytical consistency, but these measures cannot establish that all pre-analytical variation was eliminated. Spectrophotometric RNA assessment does not establish RNA integrity, and the possible influence of tissue ischemia and other incompletely characterized pre-analytical factors remains relevant. miR-1228-3p was chosen using literature and preliminary candidate testing, but its stability was not comprehensively validated across the present clinicopathological subgroups. Shared-reference normalization and residual plate variation may also have contributed to inter-miRNA correlations. Amplification efficiencies and other quality-control parameters should not be assumed to have been independently validated solely because commercial TaqMan assays were used. Finally, the study did not combine patient-matched miRNA and ADAM protein measurements or perform functional assays. Accordingly, the results identify tissue-level expression patterns and statistical associations, not direct regulatory mechanisms.

## 4. Materials and Methods

### 4.1. Characteristics of the Study Group

The analysis included 67 patients with CRC who had undergone surgery in the Department of General and Gastroenterological Surgery with the Oncological Surgery Subdivision in Bytom, Poland. This is the same study group for which the expression of selected *ADAM* genes was determined, and its detailed characteristics, and the specific exclusion criteria are described in a previous paper by Kalita et al. [28].

The expression of the selected miRNAs and *ADAM* genes was assessed in tumor tissue and in surgical margin tissue from 67 patients (*n* = 67). Rather than excluding patients with missing data across all markers, statistical analyses were performed on all available complete paired observations for each specific parameter. Valid paired tissue measurements were obtained for up to *n* = 66 patient pairs for miRNAs (miR-125b-5p: *n* = 66, miR-338-3p: *n* = 65, miR-30c-5p: *n* = 66) and up to *n* = 62 patient pairs for *ADAM* family genes (*ADAM10*: *n* = 61, *ADAM12*: *n* = 57, *ADAM17*: *n* = 62, *ADAM28*: *n* = 57). The baseline characteristics of the study cohort are summarized in Table 5.

Fundamental patient characteristics, including anthropometric data, comorbidities, the use of pharmacological agents or other stimulants, as well as imaging and histopathological findings, were extracted from the patients’ medical records. The staging of CRC was conducted in accordance with the 8th edition of the TNM Classification of Malignant Tumors, as established by the Union for International Cancer Control, utilizing the outcomes of histopathological and imaging assessments [53].

### 4.2. The Tissue Samples

Tissue specimens were collected during standard colorectal resections according to the sampling procedure described by Kalita et al. [28]. For each patient, the material comprised histopathologically confirmed colorectal adenocarcinoma and a corresponding surgical margin in which the absence of malignant cells was confirmed histopathologically. Following collection, samples were placed in sterile tubes containing RNAlater-RNA stabilization solution, subsequently frozen, transported on ice to the Department of Medical and Molecular Biology in Zabrze, Medical University of Silesia in Katowice, and stored at −80 °C until molecular analysis. The surgical margin was used as patient-matched histologically nonmalignant comparison tissue. It should not be regarded as equivalent to mucosa from cancer-free individuals. Analyses were performed on whole-tissue homogenates, without separation of epithelial, stromal, immune, and tumor-cell populations.

### 4.3. Determination of ADAM Genes Expression

*ADAM10*, *ADAM12*, *ADAM17*, and *ADAM28* transcript expression data used in the present association analyses were generated and characterized previously in this same patient cohort [28]. In that study, relative mRNA expression was determined by RT-qPCR with TaqMan™ Gene Expression Assays (Applied Biosystems, USA): ADAM10 (Hs00153853_m1), ADAM12 (Hs01106101_m1), ADAM17 (Hs01041915_m1), and ADAM28 (Hs00248020_m1). The experimental procedure and quality-control details for ADAM transcript measurement are described in the original publication [28]. No new ADAM transcript expression measurements were generated for the present study.

### 4.4. miRNAs Extraction

The tissue, with a volume of approximately 1 cm^3^ each, were homogenized using a FastPrep^®^-24 homogenizer (MP Biomedicals, Solon, CA, USA) with ceramic beads Lysing Matrix D (MP Biomedicals, Solon, CA, USA). The miRNAs were extracted using miRNeasy Tissue/Cells Advanced Mini Kit (QIAGEN, Hilden, Germany) according to the manufacturer’s instructions. The concentration and purity of the isolated RNA were determined using spectrophotometry in a NanoPhotometer Pearl UV/Vis Spectrophotometer (Implen, Munich, Germany).

### 4.5. Complementary DNA (cDNA) Synthesis

Complementary DNA (cDNA) was synthesized from total RNA (5 ng) samples using the TaqMan™ Advanced miRNA cDNA Synthesis Kit (Applied Biosystems, Foster City, CA, USA) following the manufacturer’s instructions. The procedure consisted of four reactions: the poly(A) tailing reaction, the adaptor ligation reaction, the reverse transcription (RT) reaction and the miR-Amp reaction. All reactions were performed in Mastercycler personal (Eppendorf, Hamburg, Germany).

The Poly(A) Reaction Mix, comprising 0.5 µL of 10× Poly(A) Buffer, 0.5 µL of ATP, 0.3 µL of Poly(A) Enzyme, and 1.7 µL of RNase-free water (EURx, Gdańsk, Poland), was combined with the RNA sample (2 µL) to a final volume of 5 µL. The reaction was incubated in a thermal cycler at 37 °C for 45 min, followed by a termination step at 65 °C for 10 min.

Following polyadenylation, a 5′ adaptor was ligated to the modified miRNA to serve as a forward primer binding site for downstream amplification. The Ligation Reaction Mix (consisting of 3 µL of 5× DNA Ligase Buffer, 4.5 µL of 50% PEG 8000, 0.6 µL of 25× Ligation Adaptor, 1.5 µL of RNA Ligase, and 0.4 µL of RNase-free water) was added to the poly(A) tailing product, reaching a total volume of 15 µL. The ligation was performed at 16 °C for 60 min.

The RT Reaction Mix (containing 6 µL of 5X RT Buffer, 1.2 µL of dNTP Mix, 1.5 µL of 20X Universal RT Primer, 3 µL of 10× RT Enzyme Mix, and 3.3 µL of RNase-free water), was added to the ligation product for a final volume of 30 µL. The thermal profile for reverse transcription consisted of 42 °C for 15 min, followed by enzyme inactivation at 85 °C for 5 min.

The RT product (5 µL) was combined with 45 µL of miR-Amp Reaction Mix, which included 25 µL of 2× miR-Amp Master Mix, 2.5 µL of 20× miR-Amp Primer Mix, and 17.5 µL of RNase-free water. The amplification was carried out with the following thermal cycling conditions: initial enzyme activation at 95 °C for 5 min, followed by 14 cycles of denaturation at 95 °C for 3 s and annealing/extension at 60 °C for 30 s. A final stop reaction was performed at 99 °C for 10 min. The resulting cDNA was stored at −20 °C.

### 4.6. MiRNA Selection

The target miRNAs were selected using in silico predictions of potential interactions with the investigated *ADAM* genes in miRCode (version 11) [54], miRDB (version 6.0) [55], and TargetScan (version 7.2) [56], together with published studies implicating miR-125b-5p [18,20], miR-338-3p [21,22], and miR-30c-5p [24] in CRC biology. Reports of miR-338-3p–ADAM17 interactions in gastric cancer [25,26] and miR-30c-ADAM19 interactions in CRC [27] informed this exploratory selection. However, those findings do not provide direct experimental evidence that three selected miRNAs regulate *ADAM10*, *ADAM12*, *ADAM17*, or *ADAM28* in colorectal tissue. These four *ADAM* genes were included because their expression had already been characterized in the same patient cohort [28].

### 4.7. The Relative Expression (RQ) of miRNAs

The relative expression of miR-125b-5p, miR-338-3p, and miR-30c-5p was measured using TaqMan™ Advanced miRNA Assays (Applied Biosystems, Foster City, CA, USA) on a QuantStudio 5 Real-Time PCR System, with data acquisition and analysis performed in QuantStudio Analysis Software v1.5.1. The endogenous reference miR-1228-3p was selected on the basis of published investigations of candidate reference miRNAs in CRC tissue, including the work of Danese et al. [57], and preliminary assessment of an alternative candidate in our material. MiR-520d was also evaluated preliminarily but was not consistently detectable in all examined tissue samples and therefore was not selected as the normalization reference. A common pooled calibrator was prepared from five randomly selected histologically tumor-free surgical margin samples. Following RNA isolation, RNA from these samples was adjusted to an equal concentration, and equal volumes were combined to form a single pooled calibrator. The same pooled material was used on every RT-qPCR plate. Within each analytical run, the target miRNA, miR-1228-3p, and the corresponding measurements of the common calibrator were analyzed using the same workflow. The pooled calibrator served as an inter-run technical reference for relative quantification, not as an independent biological control group.

Each qPCR reaction had a final volume of 20 µL and contained 5 µL of cDNA (diluted 1:10), 10 µL of TaqMan™ Fast Advanced Master Mix, 1 µL of the relevant TaqMan™ Advanced miRNA Assay, and 4 µL of RNase-free water. The assay IDs were 477885_mir (miR-125b-5p), 478037_mir (miR-338-3p), 478008_mir (miR-30c-5p), and 478643_mir (miR-1228-3p). Cycling conditions were 95 °C for 20 s, followed by 40 cycles at 95 °C for 1 s and 60 °C for 20 s. Investigated miRNAs were analyzed in technical duplicates. A maximum difference of 0.5 Ct between duplicate measurements was accepted. Measurements exceeding this threshold were repeated. No-template controls were included and showed no detectable amplification.

Expression of each target miRNA was normalized to miR-1228-3p (ΔCt = Ct[target miRNA] − Ct[miR-1228-3p]). The normalized value for each specimen was then compared with the corresponding normalized value of the common pooled calibrator (ΔΔCt = ΔCt[specimen] − ΔCt[calibrator]), and relative quantity (RQ) was calculated as 2^−∆∆Ct^. The same normalization and calibration approach was used across analytical batches.

### 4.8. Statistical Analysis

Statistical analyses and data visualization were performed in R version 4.4.0 (R Foundation for Statistical Computing, Vienna, Austria) within RStudio version 2025.09.1 build 401 (Posit Software, PBC, Boston, MA, USA) using the readxl [58], dplyr [59], tidyr [60], ggplot2 [61], stats [62] and rstatix [63] packages. Continuous miRNAs and *ADAM* genes expression levels are presented as median values and interquartile ranges (IQR, 25th–75th percentiles), while categorical parameters are expressed as counts and percentages (*n*, %). Data distributions were evaluated using the Shapiro–Wilk test. Paired tissue comparisons were evaluated using the Wilcoxon signed-rank test with effect sizes (*r*). Independent subgroup comparisons were performed using the Mann–Whitney U test or Kruskal–Wallis test with post-hoc Mann–Whitney U tests where appropriate. Correlations were assessed using the Spearman rank correlation coefficient (r_S_). *p*-values were adjusted using the Benjamini–Hochberg (BH) procedure [64], applied separately for each set of comparisons (paired tissue analyses, microRNA co-expression matrices, microRNA-ADAM correlation matrices, and clinicopathological subgroup analyses), with statistical significance defined as *p_adj_* < 0.05, while raw *p*-values describe nominal trends. Missing data were handled using a pairwise complete-case approach for each specific test, and exact sample sizes (*n*) are reported for each comparison. Post hoc power calculations were performed for primary paired tissue comparisons based on observed effect sizes (α = 0.05). Bivariate relationships were visualized using scatter plots featuring fitted linear regression lines with 95% confidence intervals (shaded ribbons bounded by dashed lines). Point fill colors differentiated tissue compartments (orange for tumor tissue, teal for surgical margin, and purple for paired comparisons), while regression line colors indicated the sign of the fitted slope (red for positive, blue for negative).

## 5. Conclusions

The present study suggests that alterations in miRNA expression in colorectal cancer may reflect tumor-associated molecular changes that are not readily captured by correlations with the transcript levels of potential target genes. In particular, miR-125b-5p emerges as a candidate for further investigation into the molecular processes associated with colorectal carcinogenesis, although its functional role cannot be established from tissue-level expression analysis alone. The findings also highlight the limitations of inferring miRNA-mediated regulation from bulk tissue measurements, where cellular heterogeneity and post-transcriptional mechanisms may influence the observed relationships. Further functional studies integrating miRNA, mRNA, and protein expression are needed to clarify the biological significance of these observations and the potential involvement of miRNAs in ADAM-related regulatory pathways.

## Figures and Tables

**Table 1:** Relative microRNAs expression (RQ) compared between tumor and margin tissue (*n* = 67).

miRNA	*n* (Pairs)	Tumor Median (IQR)	Margin Median (IQR)	*p*	*p_adj_*	Effect Size (*r*)
miR-125b-5p	66	0.46 (1.41)	1.33 (2.35)	**0.010**	**0.035**	0.225
miR-338-3p	65	0.95 (2.98)	1.70 (2.93)	0.305	0.375	0.090
miR-30c-5p	66	0.71 (1.57)	1.23 (1.69)	0.125	0.219	0.133

Statistically significant values are shown in bold.

**Table 2:** Correlations between selected *ADAM* gene and miRNA expression (RQ) in tumor tissue (*n* = 64–66).

*ADAM*-miRNA	*n*	r_S_	*p*	*p_adj_*
*ADAM10*-miR-30c-5p	65	0.062	0.625	0.980
*ADAM10*-miR-125b-5p	65	0.025	0.841	0.992
*ADAM10*-miR-338-3p	65	−0.083	0.510	0.980
*ADAM12*-miR-30c-5p	65	0.141	0.262	0.808
*ADAM12*-miR-125b-5p	65	0.002	0.986	0.992
*ADAM12*-miR-338-3p	65	0.019	0.882	0.992
*ADAM17*-miR-30c-5p	66	−0.037	0.766	0.980
*ADAM17*-miR-125b-5p	66	0.009	0.944	0.992
*ADAM17*-miR-338-3p	66	−0.191	0.124	0.626
*ADAM28*-miR-30c-5p	64	0.142	0.263	0.808
*ADAM28*-miR-125b-5p	64	0.145	0.253	0.808
*ADAM28*-miR-338-3p	64	0.157	0.215	0.808

**Table 3:** Correlations between selected *ADAM* gene and miRNA expression (RQ) in margin tissue (*n* = 58–63).

*ADAM*-miRNA	*n*	r_S_	*p*	*p_adj_*
*ADAM10*-miR-30c-5p	63	0.041	0.749	0.891
*ADAM10*-miR-125b-5p	63	0.125	0.328	0.736
*ADAM10*-miR-338-3p	62	−0.070	0.589	0.865
*ADAM12*-miR-30c-5p	59	−0.005	0.969	0.989
*ADAM12*-miR-125b-5p	59	0.026	0.843	0.951
*ADAM12*-miR-338-3p	58	0.088	0.512	0.848
*ADAM17*-miR-30c-5p	63	−0.099	0.441	0.846
*ADAM17*-miR-125b-5p	63	−0.049	0.701	0.874
*ADAM17*-miR-338-3p	62	−0.040	0.760	0.891
*ADAM28*-miR-30c-5p	60	0.255	0.049	0.305
*ADAM28*-miR-125b-5p	60	0.191	0.144	0.579
*ADAM28*-miR-338-3p	59	−0.010	0.943	0.986

**Table 4:** Correlations between selected miRNAs’ expression (RQ) in tumor and margin tissue (*n* = 65–67).

Tissue	miRNA-miRNA	*n*	r_S_	*p*	*p_adj_*
Margin	miR-125b-5p-miR-30c-5p	66	0.573	**<0.001**	**<0.001**
Margin	miR-338-3p-miR-30c-5p	65	0.482	**<0.001**	**<0.001**
Margin	miR-125b-5p-miR-338-3p	65	0.151	0.231	0.661
Tumor	miR-338-3p-miR-30c-5p	67	0.630	**<0.001**	**<0.001**
Tumor	miR-125b-5p-miR-30c-5p	67	0.565	**<0.001**	**<0.001**
Tumor	miR-125b-5p-miR-338-3p	67	0.443	**<0.001**	**0.002**

Statistically significant values are shown in bold.

**Table 5:** Characteristics of the study group (*n* = 67).

		*N*	(%)
All		67	
Sex	Female	34	(51)
	Male	33	(49)
Age (years)	50–65	29	(43)
	>65	38	(57)
Clinical stage	Stage I	11	(16)
	Stage II	13	(19)
	Stage III	22	(33)
	Stage IV	21	(31)
Pathomorphological stage			
*T parameter* (*n* = 66)	T1	2	(3)
	T2	12	(18)
	T3	28	(42)
	T4	24	(36)
*Nodal status* (*n* = 67)	N0	25	(37)
	N1	19	(29)
	N2	23	(34)
*Metastasis* (*n* = 66)	M0	45	(67)
	M1	21	(31)
Histological grade (*n* = 66)	G1	3	(4)
	G2	52	(78)
	G3	11	(16)
Tumor location	Colon (non-rectal)	45	(67)
	Rectum	22	(33)
Sidedness (*n* = 66)	Right-sided colon	23	(34)
	Left-sided colon	43	(64)
Neoadjuvant therapy (*n* = 65)	Yes	11	(16)
	No	54	(81)
BMI (kg/m^2^)	Normal weight (18.5–24.9)	20	(29)
	Overweight (25.0–29.9)	29	(43)
	Obesity (≥30.0)	18	(27)
Cardiovascular disease		45	(67)
Stimulants		17	(25)
	Nicotine	14	(21)
	Alcohol	3	(4)

Percentages may not sum to 100% due to minor missing clinical data in selected categories. Missing clinical data were present for T parameter (*n* = 1), metastasis (*n* = 1), histological grade (*n* = 1), and neoadjuvant therapy (*n* = 2). Sidedness classification excludes one transverse colon tumor (*n* = 66).

## Data Availability

The data presented in this study are available on request from the corresponding author. The data are not publicly available due to the protection of patient data.

## Abbreviations

3′UTR	3′ untranslated region
ADAM	a disintegrin and metalloproteinase (adamalysin)
AKT	protein kinase B
ATP	adenosine triphosphate
AXIN2	axin 2
BH	Benjamini–Hochberg (procedure)
BMI	body mass index
CAF	cancer-associated fibroblast
cDNA	complementary DNA
CFTR	cystic fibrosis transmembrane conductance regulator
CGN	cingulin
CI	confidence interval
circRNA	circular RNA
CRC	colorectal cancer
CVD	cardiovascular disease
DNA	deoxyribonucleic acid
dNTP	deoxynucleoside triphosphate
EGF	epidermal growth factor
EMT	epithelial–mesenchymal transition
IGF	insulin-like growth factor
IQR	interquartile range
lncRNA	long non-coding RNA
*MACC1*	metastasis-associated in colon cancer 1
MAPK	mitogen-activated protein kinase
MCL1	myeloid cell leukemia 1
MiRNA, miR	microRNA
mRNA	messenger RNA
MYC	MYC proto-oncogene
NF-κB	nuclear factor kappa B
*p_adj_*	*p*-value adjusted for multiple comparisons (Benjamini–Hochberg)
PCR	polymerase chain reaction
PDCD4	programmed cell death 4
PEG	polyethylene glycol
PI3K	phosphoinositide 3-kinase
PTEN	phosphatase and tensin homolog
Q1, Q3	first and third quartile
RT-qPCR	quantitative real-time polymerase chain reaction
RAS	rat sarcoma viral oncogene homolog
RhoA	Ras homolog family member A
RISC	RNA-induced silencing complex
RNA	ribonucleic acid
RNase	ribonuclease
ROCK	Rho-associated coiled-coil containing protein kinase
RQ	relative quantification (relative expression)
r_S_	Spearman’s rank correlation coefficient
RT	reverse transcription
SMO	smoothened
*STAT3*	signal transducer and activator of transcription 3
TAM	tumor-associated macrophage
TCF7	transcription factor 7
TGF-β	transforming growth factor beta
TNM	tumor, node, metastasis (classification)
TRAF6	TNF receptor-associated factor 6
USP22	ubiquitin-specific peptidase 22
WGCNA	weighted gene co-expression network analysis
